# Associations between serum trace elements and inflammation in two animal models of nonalcoholic fatty liver disease

**DOI:** 10.1371/journal.pone.0243179

**Published:** 2020-12-11

**Authors:** Giuseppina Palladini, Andrea Ferrigno, Laura Giuseppina Di Pasqua, Clarissa Berardo, Vittoria Rizzo, Stefano Perlini, Mariapia Vairetti

**Affiliations:** 1 Dept of Internal Medicine and Therapeutics, University of Pavia, Pavia, Italy; 2 Fondazione IRCCS Policlinico San Matteo, Pavia, Italy; 3 Dept of Molecular Medicine, University of Pavia, Pavia, Italy; 4 Emergency Department, Fondazione IRCCS Policlinico San Matteo, Pavia, Italy; Fudan University, CHINA

## Abstract

**Background:**

The comparison of hepatic steatosis animal models has allowed the understanding of mechanisms involved in the pathogenesis of non-alcoholic fatty liver disease (NAFLD) and the progression to nonalcoholic steatohepatitis (NASH). We investigated the changes in serum levels of trace elements and inflammation markers in fatty livers using two rat models of NAFLD, the methionine and choline deficient (MCD) diet model and Obese-Zucker rats.

**Material and methods:**

NAFLD was induced in male Wistar rats by 3-week MCD diet administration, after which, blood samples were collected. 12-week old Obese (fa/fa) and Lean (fa/-) male Zucker rats were also used. Serum levels of hepatic enzymes, Urea, Uric acid, Ca^2+,^ Cl, Fe, K, Na, Mg and Zn were quantified, as well as the inflammation markers TNF-alpha, IL-1beta and IL-6.

**Results:**

In MCD rats, a serum increase in Cl, Mg and Na and a decrease in Ca^2+,^ Zn were detected in comparison with control rats. An increase in only serum Ca^2+^ was found in Obese-Zucker rats. In MCD rat serum, Zn was inversely correlated with IL-1beta, IL-6, TNF-alpha, Urea and Uric Acid; Ca^2+^ was inversely correlated with IL-1beta, IL-6 and Urea; Cl and Mg were directly correlated with Uric Acid and Urea, respectively. In Obese-Zucker rats, Cl and IL-1beta were inversely correlated, whereas Ca^2+^ and Urea where directly correlated, as well Fe and TNF-alpha.

**Conclusions:**

The serum concentrations of trace elements change significantly only in MCD rats, which spontaneously progress to NASH. The causes of these changes may be a result of defense strategies of the organism, which is regulated by immunoregulatory cytokines. These results might suggest that the impairment of trace element status should be taken into account when the effectiveness of a pharmacological treatment is under evaluation.

## Introduction

Non-alcoholic fatty liver disease (NAFLD) is a rapidly growing public health problem representing the most common liver disease [1]. Currently, the prevalence of NAFLD is estimated between 25 and 45% and the main risk factors are metabolic, including obesity, hyperlipidemia, hypertension, insulin resistance (IR), and type2 diabetes mellitus (T2DM) [1].

NAFLD is a progressive disease whose occurrence and development are complex and still unclear. A growing body of evidence from clinical and experimental studies indicates that impairment of trace elements status occurred in pathological conditions such as NAFLD. In particular, changes in trace elements have been reported in plasma of NAFLD patients: Zn exhibited a negative association with the severity of the disease thus higher plasma Zn levels were associated with lower severity of NAFLD [2]. In addition, serum Ca^2+^ levels is significantly associated with NAFLD [3]. Furthermore, NAFLD significantly affects trace element status in experimental animals: serum levels of I, Li, and Mn were significantly lower whereas serum levels of Co, Se, V, and Sr exceeded the control values [4]. However, the impact of NAFLD on trace element serum concentration remains unclear.

Due to a number of studies reporting an association between elevated serum uric acid levels and the development of NAFLD, uric acid has been proposed to play a crucial role in the pathogenesis of hepatic steatosis. In addition, serum uric acid recently emerged as possible predictor of NAFLD [5].

Preclinical animal models are indispensable to identify novel drug targets for the developments of future therapies. The experimental models used for the identification of the NAFLD pathogenesis and its treatment by pharmacological molecules including natural product [6], can be classified into genetic and nutritional models. The most relevant genetic model is obese Zucker rat (fa/fa), while the most commonly used nutritional model employs a methionine-choline-deficient diet (MCD diet). Unfortunately, each model presents strengths and weaknesses with regard to their comparability to human disease conditions [7,8].

Zucker rats, (Leprfa/Leprfa (fa/fa) have a natural mutation in the leptin receptor (fa allele), which decreases the affinity of the receptor for leptin and alters signal transduction. Zucker rats spontaneously develop severe obesity, insulin resistance and steatosis and are hyperleptinemic, hyperphagic and inactive. These rats do not spontaneously develop NASH [9]. Genetic models have some advantages regarding the experimental duration and severity of NAFLD, while retaining the metabolic characteristics associated with NAFLD. However, the limitation of a genetic model is that these mutations are very rare in humans.

The methionine and choline deficient diet (MCD) is one of the best used dietary patterns for NAFLD [10]. The lack of essential nutrients such as choline and methionine leads to reduced β-oxidation and reduced production of very low density lipoprotein particles (VLDL) [11]. Choline deficiency causes impaired hepatic secretion of VLDL, resulting in hepatic fat accumulation, liver cell death, oxidative stress and changes in cytokines and adipokines, accompanied by minor inflammation and fibrosis [12]. Methionine deficiency leads to more evident inflammation and early development of fibrosis (after 8–10 weeks) [13]. Limit of this model is the lack of metabolic features seen in human NAFLD, including obesity, peripheral insulin resistance, and dyslipidemia.

Therefore, using both a genetic and nutritional experimental model of NAFLD, the aims of this study have been to (a) determine the serum levels of trace elements (Ca^2+^, Cl, Fe, K, Na, Mg and Zn), and (b) evaluate a potential relationship between these trace elements, inflammatory markers (TNF-alpha, IL-1beta and IL-6) and uric acid.

## Materials and methods

All reagents were obtained from SIGMA (Italy) and were of the highest grade of purity available.

### Animals

The animal models used were approved by the Italian Ministry of Health and the Pavia University Animal Care Commission (Document number 2/2012). The study was carried out in strict accordance with the Guidelines For the Care and Use of Laboratory Animals (University of Pavia, Pavia, Italy). Rats were housed under specific pathogen-free conditions with 12:12 light:dark cycle at 22±2°C and 60±5% humidity. Sterile water and chow were available ad libitum.

Both Wistar and Zucker rats were purchased from Charles River, Italy. Two animal models of NAFLD were used: 1) male Wistar rats (8 week old), fed with either a methionine-choline deficient diet (n = 7) or a isocaloric control diet (n = 8) (Piccioni, Italy) for 3 weeks; 2) Obese (fa/fa) (n = 6; 375±15 g) and Lean (fa/-) (n = 3; 300±10 g) 11-week old male Zucker rats. The animal health was daily monitored evaluating the indices of stress, such as: excessive grooming, apathy, aggression, chromodacriorrea, excessive reactivity to environmental stimuli, persistent chewing, matted hair. When a sign of discomfort and stress was detected, the administration of the diet has been interrupted. No mortality occurred during this planned study.

In order to comply with the principle of the three Rs (Replacement, Reduction, Refinement) for care and use of animals, attention was paid to: i) standardizing the procedures, performed by the same operator to avoid operator variability, ii) minimizing the animal suffering, iii) minimizing the number of animals; iv) obtaining the maximum information from each animal.

### Blood sampling and analysis

At the time of sacrifice, blood samples were collected from vena cava and the serum was snap frozen in liquid nitrogen. Liver injury was assessed by serum levels of alanine transaminase (ALT), aspartate transaminase (AST) and alkaline phosphatase (ALPH). These parameters and Urea, Uric acid, K, Na, Fe, Ca^2+^ and Cl were analyzed by an automated Hitachi 747 analyzer (Roche/Hitachi, Indianapolis, IN, USA). Serum levels of Mg and Zn were evaluated by Abbott ARCHITECT system (Abbott Laboratories, Chicago, Illinois, USA). Serum levels of inflammation markers, TNF-alpha (Cod 865000192), IL-1beta (Cod 670040096) and IL-6 (Cod 670010096) were determined by using ELISA Kits (Bertin Bioreagent).

### Histological analysis

Liver samples were quickly removed from MCD and Zucker rats and snap frozen in liquid nitrogen. Frozen sections (8 *μ*M thick) were collected on glass slides and stored at −80° C until staining with Hematoxylin and Eosin (H&E).

### Statistical analysis

Normal data distribution was analyzed by Kolmogorov-Shapiro normality test. Statistical analysis was performed with one-way ANOVA, followed by Tukey’s multiple comparisons test. When data distribution was not normal Kruskall-Wallis and Dunn’s test was used. The correlation analysis was evaluated according to Pearson correlation for normally distributed variables or Spearman rank correlation when the variables are not normally distributed. The value of p<0.05 was considered to indicate statistical significance. The accompanying tables present the mean value ± standard error of the mean (SEM). Statistical analysis was performed using MedCalc Statistical Software version 18.11.3.

## Results

The evaluation of trace element serum levels showed a significant increase in Cl, Na and Mg and a significant decrease in Ca^2+^ and Zn in MCD rats when compared with their control rats (Table 1). Only a significant increase in Ca^2+^ was found in serum of Obese Zucker rats when compared with their control Lean rats (Table 1). A comparison of the two animal models showed lower levels in Ca^2+^ and Zn as well as higher levels in Cl and Mg in MCD versus Obese Zucker rats. Comparable levels of K, Na and Fe ware detectable in these two models of NAFLD (Table 1).

**Table 1:** Serum elements in MCD and Obese Zucker rats.

	Control MCD	MCD	*p*^1^	Lean Zucker	Obese Zucker	*p*^1^	*p*^2^
**Ca**^**2+**^	10.62 ±0.20	**9.84 ±0.29**	<0.001	10.77±0.07	**12.12±0.22**	<0.001	**<0.001**
**Cl**	101.8 ±1.07	**105.2 ±0.73**	<0.05	102.7±1.20	**100.25±0.7**	ns	**<0.05**
**Fe**	191.8 ±10.45	**249.40 ±36.94**	ns	300.7±76.4	**296.0±33.5**	ns	**ns**
**K**	4.10 ±0.29	**4.77 ±0.22**	ns	4.47±0.13	**4.90±0.20**	ns	**ns**
**Na**	140.40 ±2.18	**145.40 ±1.36**	<0.05	147.3±0.88	**147.7±0.2**	ns	**ns**
**Mg**	1.64 ±0.05	**1.84 ±0.07**	<0.05	1.60±0.019	**1.46±0.07**	ns	**<0.05**
**Zn**	93.00 ±3.56	**59.60 ±5.95**	<0.001	176.2±11.5	**173.1±11.1**	ns	**<0.001**

Na, K and Cl: mEq/l; Ca^2+^ and Mg; mg/dl; Fe and Zn: Mcg/dl. p^1^: Control vs MCD or Lean vs Obese; p^2^ MCD *vs* Obese; ns: p value not significant.

**Fig 1:**
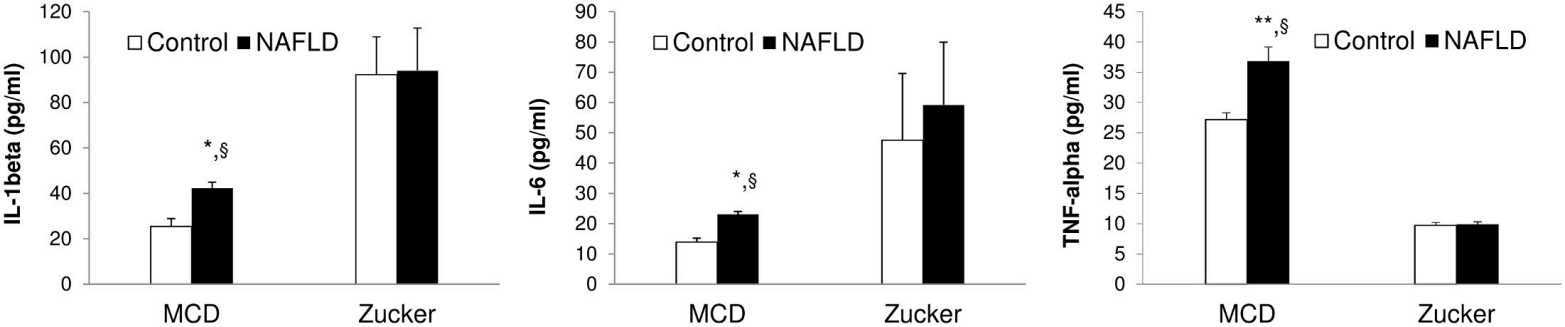
Serum levels of inflammation markers in MCD and Obese Zucker rats. Serum levels of IL-1beta (pg/ml), IL-6 (pg/ml) and TNF-alpha (pg/ml) were determined in two animal models of NAFLD: 1) male Wistar rats fed with either a methionine-choline deficient diet (MCD) or a isocaloric control diet (Piccioni, Italy) for 3 weeks; 2) Obese (fa/fa) and Lean (fa/-) 11-week old male Zucker rats. *p<0.05 and **p<0.001: MCD vs Control; ^§^MCD vs Obese Zucker. §p<0.05: MCD vs Obese Zucker.

Serum levels of ALT, AST, ALPH and urea did not present statistically significant differences when comparing MCD with Obese Zucker rats (Table 2). Lower levels of IL-1beta and IL-6 and higher levels of TNF-alpha and uric acid were found in MCD versus Obese Zucker rats (Fig 1). An increase in ALT, AST, ALPH, Urea, Uric acid, IL-beta, IL-6 and TNF-alpha were found in serum of MCD rats when compared with their control rats (Table 2 and Fig 1). Comparable levels of AST, ALPH, IL-1beta, IL-6 and TNF-alpha were found in Obese Zucker rats when compared with their control rats (Table 2 and Fig 1). An increase in ALT and Urea as well as a decrease in uric acid were found in serum of Obese Zucker rats when compared with their control rats (Table 2).

**Table 2:** Serum parameters in MCD and Obese Zucker rats.

	Control MCD	MCD	*p*^1^	Lean Zucker	Obese Zucker	*p*^1^	*p*^2^
**ALT**	24.4±1.4	**136.40±38.7**	*<0*.*001*	71.0±3.0	**116.80±18.6**	*<0*.*05*	**ns**
**AST**	57.00±5.0	**134.20±19.1**	*<0*.*05*	112.00±3.0	**114.6±10.2**	*ns*	**ns**
**ALPH**	128.80±6.3	**198.00**±27.5	*<0*.*05*	194.50±6.5	**196.75**±13.4	*ns*	**ns**
**Urea**	30.4±4.8	**54.80±1.8**	*<0*.*001*	45.0±1.5	**64.6±4.9**	*<0*.*01*	**ns**
**Uric Acid**	1.0±0.06	**1.26±0.12**	*<0*.*05*	1.17±0.03	**0.97±0.06**	*<0*.*05*	**<0.05**

ALT, AST and ALPH: mU/ml; Urea and Uric Acid: mg/dl. p^1^:Control vs MCD or Lean vs Obese; p^2:^ MCD *vs* Obese;.ns: p value not significant.

**Fig 2:**
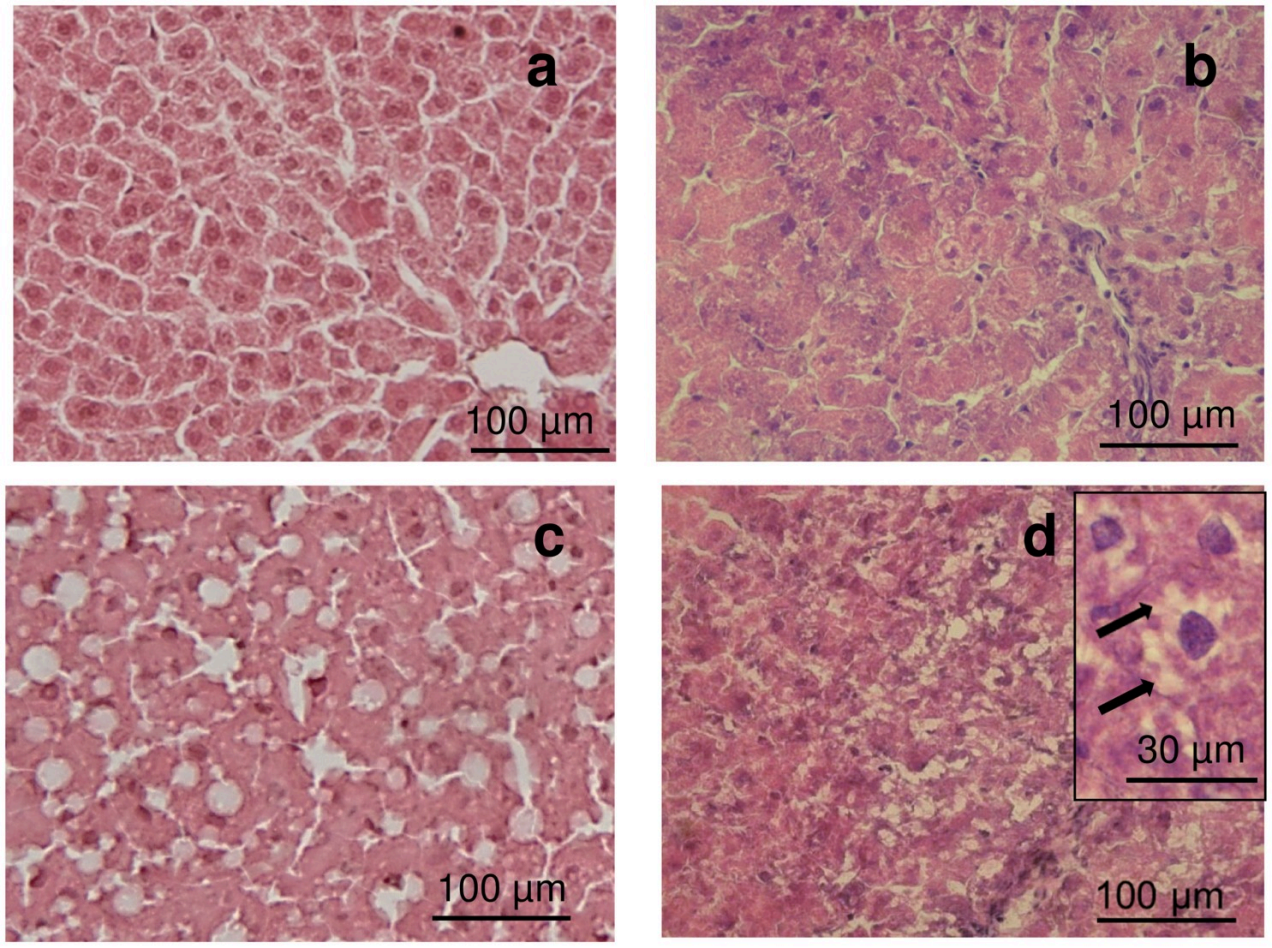
The representative photomicrographs present the histopathological alterations found in MCD and Zucker rats. (a) control MCD and (b) Lean Zucker: Control livers, the histological appearance is normal; (c) MCD rats present macrovesicular steatosis; (d) Obese Zucker rats present microvesicular fatty infiltration (arrows) (Hematoxylin & Eosin staining).

We evaluate the hepatocellular steatosis in tissue samples obtained from MCD and obese Zucker rats as shown in Fig 2. The histological appearance was normal in the control diet group (Fig 2a) while macrovesicular steatosis was the main feature of steatosis after 3 weeks of MCD diet (Fig 2b).

Whereas lean Zucker rats livers appeared normal (Fig 2c), the livers of obese Zucker rats showed hepatocellular injury characterized by microvesicular fatty infiltration and ballooning degeneration (Fig 2d).

**Fig 3:**
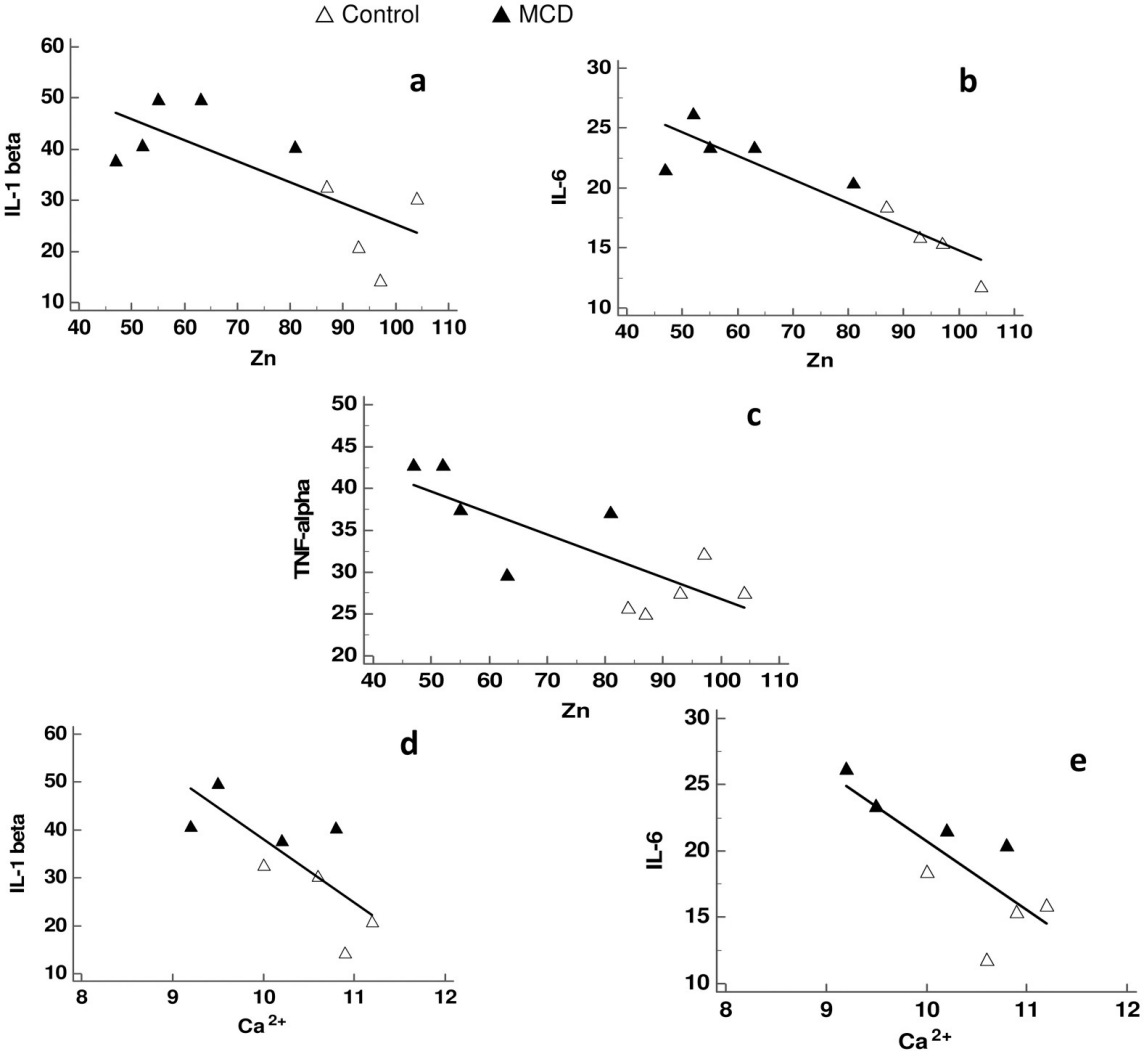
Correlation between proinflammatory markers versus Zn and Ca^2+^ in MCD rats. (a-c): Zn versus IL-1beta, IL-6 and TNF-apha; (d-e): Ca^2+^ versus IL-1beta and IL-6.

In MCD rats, correlation analysis showed that serum Zn levels were strongly and inversely correlated with IL-1beta, IL-6, TNF-alpha (Table 3, Fig 3). The same trend occurred for Urea and Uric Acid. Furthermore, serum Ca^2+^ levels were also inversely correlated with IL-1beta and IL-6 (Table 3, Fig 3). The same trend occurred for Urea. Cl and Mg serum levels were found to be inversely correlated only with Uric Acid and Urea, respectively. No correlation was found between Fe and all the parameters taken into account (Table 3).

**Table 3:** Correlation between serum trace elements and inflammatory markers in MCD rats.

	Zn		Cl		Ca^2+^		Mg		Fe	
	r/r_s_	*p*	r/r_s_	*p*	r/r_s_	*p*	r/r_s_	*p*	r/r_s_	*p*
**IL-1beta**	**-0.717 r**_**s**_	<0.05	**0.522 r**_**s**_	ns	**-0.812 r**_**s**_	<0.05	**0.552 r**_**s**_	ns	**0.183 r**_**s**_	ns
**IL-6**	**-0.911 r**_**s**_	<0.001	**0.419 r**_**s**_	ns	**-0.812 r**_**s**_	<0.05	**0.182 r**_**s**_	ns	**0.083 r**_**s**_	ns
**TNF-alpha**	**-0.766 r**_**s**_	<0.05	**0.528 r**_**s**_	ns	**-0.336 r**_**s**_	ns	**0.420 r**_**s**_	ns	**0.305 r**_**s**_	ns
**Urea**	**-0.784 r**	<0.05	**0.439 r**	ns	**-0.794 r**	<0.05	**0.634 r**	<0.05	**0.423 r**_**s**_	ns
**Uric Acid**	**-0.639 r**_**s**_	<0.05	**0.738 r**_**s**_	<0.05	**-0.498 r**_**s**_	ns	**0.571 r**_**s**_	ns	**-0.252 r**_**s**_	ns

r: Pearson’s correlation coefficient; rs: Spearman’s rank correlation coefficient; ns: p value not significant.

In Zucker Obese rats, Cl and IL-1beta were found to be inversely correlated; Ca^2+^ and Fe were directly correlated with Urea and TNF-alpha, respectively (Table 4).

**Table 4:** Correlation between serum trace elements and inflammatory markers in Obese Zucker.

	Zn		Cl		Ca^2+^		Mg		Fe	
	r/r_s_	*p*	r/r_s_	*p*	r/r_s_	*p*	r/r_s_	*p*	r/r_s_	*p*
**IL-1beta**	**0.047 r**_**s**_	ns	**-0.789 r**_**s**_	<0.05	**-0.095 r**_**s**_	ns	**-0.400 r**_**s**_	ns	**0.001 r**_**s**_	ns
**IL-6**	**0.095 r**_**s**_	ns	**0.477 r**_**s**_	ns	**-0.059 r**_**s**_	ns	**-0.400 r**_**s**_	ns	**0.007 r**_**s**_	ns
**TNF-alpha**	**-0595 r**_**s**_	ns	**-0.257 r**_**s**_	ns	**-0.287 r**_**s**_	ns	**-0.400 r**_**s**_	ns	**0.786 r**_**s**_	<0.05
**Urea**	**-0.073 r**	ns	**-0.414 r**	ns	**0.819 r**	<0.05	**-0.770 r**	ns	**-0.146 r**_**s**_	ns
**Uric Acid**	**-0.355 r**_**s**_	ns	**0.126 r**_**s**_	ns	**-0.665 r**_**s**_	ns	**0.755 r**_**s**_	ns	**0.079 r**_**s**_	ns

r: Pearson’s correlation coefficient; rs: Spearman’s rank correlation coefficient; ns: p value not significant.

In MCD rats, correlation analysis of serum Uric Acid versus IL-1beta, IL-6, TNF-alpha, showed that only IL-6 and TNF-alpha were positive correlated; on the contrary, no correlation was found in Obese Zucker rats (Table 5).

**Table 5:** Correlation between Serum Uric Acid (SUA) and inflammatory markers in MCD and Obese Zucker rats.

	SUA MCD		SUA Obese Zucker	
	r_s_	*p*	r_s_	*p*
**IL-1beta**	**0.587**	ns	**0.252**	ns
**IL-6**	**0.683**	<0.05	**-0.189**	ns
**TNF-alpha**	**0.658**	<0.05	**0.373**	ns

rs: Spearman’s rank correlation coefficient; ns: p value not significant.

## Discussion

Our results show that NAFLD significantly affects trace element (Zn, Cl, Fe, Mg, Zn) serum levels in two experimental animals model, in agreement with precedent studies [4]; moreover, using serum samples obtained from MCD and Obese Zucker rats, the current study documented the upregulation of IL-1beta, IL-6, TNF-alpha as reported by Ji et al. [14], as well as the existence of significant correlations between these trace elements and pro-inflammation markers such as: TNF-alpha, IL-1beta and IL-6. NAFLD is the most common cause of chronic liver disease and the activation of inflammatory pathways could contribute to disease pathogenesis. Metabolic NAFLD complications, obesity, insulin resistance and type 2 diabetes involve a chronic “low-grade inflammation”, resulting in the increase in obesity-related inflammatory molecules, such as: TNF-alpha, IL-1beta, IL-6, IL-1Ra, and C-reactive protein (CRP) [15]. However, the concentration of trace elements and their relationship with pro-inflammatory cytokines in NAFLD has not yet been well documented.

TNF-alpha is a key factor in the development of NAFLD and NASH in both humans and animals and a relationship between TNF-alpha expression and insulin resistance in NASH has been previously demonstrated [16]. Indeed, in several rodent models of obesity, TNF-alpha expression in adipose tissue was upregulated as compared with controls [17,18].

The role of IL-6 in NAFLD, which is closely associated with obesity and insulin resistance, remains controversial. The multifunctional cytokine IL-6 is clearly involved in the regulation of metabolism, with confirmation of the link between obesity and inflammation [19,20]. The release of IL-6 into the systemic circulation, and the fact that this release was observed to be greater in obese Zucker rats, lends support to a recent suggestion that IL-6 plays a role as a systemic regulator of body weight and lipid metabolism [21].

Pro-inflammatory cytokines contribute to NAFLD development from liver steatosis to NASH and hepatic fibrosis. IL-1beta participates in the development and progression of NAFLD. IL-1beta is secreted as an inactive molecule needing a proteolytic cleavage to become bioactive. Mechanisms activating IL-1beta include the classical NLRP3 inflammasome-caspase-1 and the neutrophil serine proteases, neutrophil elastase, and proteinase-3 [22].

An important step in preclinical NAFLD research is the selection of the appropriate animal model. We have used the genetic Zucker Obese fa/fa model and the nutritional MCD diet model. In the present study, Zucker Obese rat develop steatosis characterized by hepatic microvesicular and fatty infiltration but it is known do not progress to NASH [23]. The MCD diet showed a typical macrovesicular steatosis as main feature at 3 weeks [24]. This last model has been widely used to study the onset and progression of NAFLD to NASH. MCD diet rapidly induces liver steatosis and inflammation which is followed by significant tissue injury and fibrosis [8]. In contrast to the genetic Zucker Obese rat model, the MCD diet does not induce insulin resistance in rats. Thus, we suggest that the reported changes in specific trace elements, already detectable in the early period of treatment, may contribute to the development of severe NASH observed only in MCD rats and not in Obese fa/fa animals.

### Zn and NAFLD

In this study, we found that serum zinc concentrations were significantly lower in MCD animals when compared with their respective control group. It has been demonstrated that changes in plasma essential trace elements, such as selenium, copper, zinc, and iron, are part of the defense strategies of an organism and are induced by IL-1beta, TNF-alpha, and IL-6 [25]. In our data, there are significant inverse correlations between Zn and IL-1beta, IL-6 and TNF-alpha. It is well known that plasma zinc concentrations decrease in NAFLD but the causes of this depletion is not yet known [26]. Although there are few reports [27] about the association between zinc and interleukins in NAFLD, the decreased concentrations of serum zinc in our MCD animals might be attributed to an inverse relationship between hepatic zinc content and plasma zinc levels [28] as a defense mechanism possibly modulated by interleukins. It has been demonstrated that interleukins, when released from activated phagocytic cells, reduce plasma zinc concentrations in experimental animals by redistributing zinc from plasma to the liver in infectious disease [29]. Zinc deficiency caused by chronic liver disease also initiates insulin resistance, iron overload, and hepatic steatosis [30]. Our data reported a not significant decrease in all serum elements. Only a significant increase in Ca^2+^ was found in the serum of Obese Zucker rats when compared with their control Lean rats. As reported by Staniek et al., [31] data obtained by tissue analysis in Zucker obese rats showed a reduction in Cu, Zn, Fe and Mg concentration in the liver, when compared with control Lean animals, presumably as a result of the increased liver fat content in Zucker Obese rats. The same is likely to happen in our serum as well. Limited number of studies have examined metal ion concentrations in Zucker Obese rats [32,33] and the role of these elements has not yet been well documented in this animal model.

### Calcium and NAFLD

Serum Calcium concentration was found to be lower in MCD rats and significant inverse correlations were also found between serum calcium and IL-1beta, IL-6 and TNF-alpha levels. A study reported evidences about the association of serum calcium levels with NAFLD [3]. The association between high calcium and NAFLD has not yet been clarified. Some studies suggests that serum calcium levels are related to hypertension [34], abnormal glucose metabolism, dyslipidemia [35] and metabolic syndrome [36]. Calcium levels could contribute to the development and progression of NAFLD by inducing mitochondrial dysfunction and oxidative stress [3]. Calcium signaling has an important role in mitochondrial energy metabolism: an increase in calcium may disrupt mitochondrial β-oxidation and increase reactive oxygen species (ROS), facilitating fat accumulation and inflammation in the liver. Indeed, in our MCD-fed rat NAFLD model, we also documented an inverse correlation between calcium and interleukins and TNF-alpha.

### Iron and NAFLD

It is most widely accepted that a modest degree of iron overload is associated with NAFLD liver injury progression, although the mechanisms by which this might occur remain unclear [37]. Liver plays a central role in iron metabolism as it is the principle source of hepcidin, the regulatory peptide hormone of iron homeostasis. In response to several stimuli such as excessive iron deposits, inflammatory signals (IL-6) or ER-stress, hepcidin is overexpressed determining a reduction in iron intestinal absorption and an increase in iron retention from macrophage and hepatocytes [38]. In addition, the systemic inflammatory state induced by NAFLD may predispose the organism to increased hepcidin levels. Serum iron levels previously reported in rats fed with the MCD diet, were comparable with those found in the MCD rats used in this study [39]. Furthermore, no significant increase in serum iron documented in the present study was comparable with results previously reported: significantly higher levels in serum iron occurred only after 4 weeks [39]. The lack in serum Fe alterations may be attributed to early NAFLD stage; this result could also explain the absence of significant correlations versus pro-inflammatory markers.

### Magnesium and NAFLD

In the present study, we found a significant hypermagnesemia in MCD rats versus control animals, whereas we detected only a modest hypomagnesemia using Obese Zucker rats. In addition, no significant correlation was found in both models between Mg and interleukins. A recent study showed, in human patients, that a lower serum magnesium concentration was independently associated with biopsy-proven hepatic steatosis and steatohepatitis [40]. Thus, a decrease in serum magnesium levels may be involved in the pathogenesis of NAFLD. In fact, an appropriate magnesium intake is linked to a reduced risk of metabolic syndrome and insulin resistance. Our data show a contrary trend that may be justified by the early NAFLD stage of our MCD model.

### Chlorine and NAFLD

Electrolytes (ie, sodium, chloride, and potassium) and minerals are inorganic compounds required for tissue structure, pH regulation, and enzymatic activities [28]. The role of electrolyte homeostasis is not well established in NAFLD. Blood pH values are critical for normal cell and organ function [41,42]. The liver, together with kidneys and lungs, is an important acid-base regulating organ, which plays a crucial role in various homeostatic pathways [43]; consequently, advanced chronic liver disease can result in a variety of acid-base disorders. The significant increase in serum levels of Cl and Na, found only in serum of MCD rats and not in Zucker Obese animals, probably reflects the tissue injury caused by the progression of NAFLD toward NASH.

### Uric Acid and NAFLD

In last years, among different serum markers considered, serum uric acid has been evaluated as possible predictors of NAFLD [38]. Two different meta-analyses of prospective studies have been shown a significant higher risk for NAFLD in subjects with higher serum uric acid [44,45]. We found higher serum uric acid in MCD rats with respect to Obese Zucker rats. We also found an inverse correlation between serum uric acid and serum levels of Zn in MCD rats, whereas a positive correlation versus TNF-alpha was observed. Some *in vitro* and *in vivo* studies reported that uric acid stimulates the synthesis of IL-6 and alpha, which are known to enhance oxidative stress [46,47]. Uric acid reduces oxidative stress [48] thus allowing to speculate that there is a regulation between cytokines and antioxidant uric acid. High levels of pro-oxidant IL-6 and TNF-alpha are associated with an uric acid increase as a compensatory mechanism to reduce oxidative stress; whereas a reduction in IL-6 and TNF-alpha levels would also result in a decrease in uric acid because of the lack of IL-6 and TNF-alpha-induced pro-oxidant stimulation [49].

Although a progress is seen in understanding of the NAFLD pathogenesis and identifying therapeutic targets, the slowest advancement is seen in the therapeutic field. The implication of this study is connected with the evaluation of the effectiveness of pharmacological treatments, including natural products which are commonly used in clinical practice [6]

### Study limitations

The limit of this study resides in evaluating trace elements only in NAFLD, avoiding the progression to NASH. Our results show, using two animal models, that a serum perturbation in some trace elements occurred thus suggesting their likely synergistic action: these serum changes may be included in a wider perspective together with other metabolic and biochemical parameters, in order to predict liver damage associated to NAFLD.

## Conclusion

We demonstrated that the serum concentrations of trace elements taking into account change significantly only in MCD diet-induced NAFLD model. The causes of these changes may not be a result of a specific deficiency from dietary inadequacies or imbalances, but may be a result of defense strategies of the organism, which are regulated by immunoregulatory cytokines. These results might also suggest that the disturbance in trace element metabolism should be taken into account when the effectiveness of a pharmacological treatments, including natural products which are commonly used in clinical practice, is under evaluation.

## Supporting information

S1 File (PDF)
