# Supplementary material for: Associations between serum trace elements and inflammation in two animal models of nonalcoholic fatty liver disease
Source: PLoS One. 2020 Dec 11;15(12):e0243179. doi: 10.1371/journal.pone.0243179 (PMC7732075; doi:10.1371/journal.pone.0243179)
Supplement: S1 File — (PDF) [file pone.0243179.s001.pdf]

TREATMENT	ALT	AST	ALPH	UREA	UricAcid	IL_1	IL_6	TNF	Ca2+	Cl	Fe	K	Na	Mg	Zn
Control	24	49	151	24	1,1	14,501	15,436	32,33	10,9	103	202	4,1	145	1,59	97
Control	19	51	127	49	0,9	32,869	18,52	25,082	10	101	207	3,57	137	1,52	87
Control	20	47	160	28	0,9			25,832	10,4	98	208	3,82	134	1,57	84
Control	27	66	132	29	1,1	30,534	11,842	27,63	10,6	103	190	4,9	145	1,5	104
Control	26	72	112	22		21,084	15,937	27,63	11,2	104	152		141	1,54	93
Control	26	72	116					25						1,71	
Control	23	65	105			28,45	11,147							1,88	
Control	30	34	126				10,385							1,84	
MCD	212	166	356	54	1	40,58	20,503	37,2	10,8	106	261	4,8	147	1,86	81
MCD	274	233	175	49	1,4	37,89	21,623	42,9	10,2	106	262	4,5	146	1,64	47
MCD	65	116	128	60	1,13	40,893	26,243	42,9	9,2	103	175	4,33	141	1,71	52
MCD	134	110	144	56	1,11	49,873	23,463	37,6	9,5	104	375	4,6	144	1,74	55
MCD	59	84	213	55	1,65	49,874	23,464	29,8	9,5	107	174	5,61	149	2,1	63
MCD	75	96	174					30,8						2,11	
MCD	136	135	196											1,72	
Obese	123	132	239	58	1,1	98,94	55,785	11,728	12,5	99	391	4,6	148	1,387	145
Obese	116	88	196	59	1,2	90,518	27,1303	10,361	11,4	101	279	4,7	147	1,53	161,5
Obese	123	150	169	63	0,9	63,405	118,629	10,121	11,9	102	233	5,5	148	1,45	210
Obese	187	123	231	59	0,9	161,798	35,3968	10,001	12,2	99	281	4,8	148		184
Obese	68	96	161	84	0,9	55,921	5,0863	8,549	12,6						165
Obese	84	96	184		0,8			9							
Lean	74	107	195	44	1,2	73,743	38,7034	10,663	10,7	102	453	4,6	146	1,62	155
Lean	65	112	205	43	1,2	125,603	14,455	8,99	10,9	101	215	4,6	147	1,582	194,4
Lean	74	118	183	48	1,1	77,453	89,4046	9,553	10,7	105	234	4,2	149	1,601	179,2
